# Transfer of peripersonal space to a virtual body in young adults and exploration of adult age differences

**DOI:** 10.1007/s00221-025-07132-6

**Published:** 2025-07-25

**Authors:** Dariusz O’Leary, Yichen Fan, Ina Schattenberg, Andrea Serino, Shu-Chen Li

**Affiliations:** 1 6G-life Research Hub, TU Dresden, Dresden, Germany; 2 Chair of Lifespan Developmental Neuroscience, Faculty of Psychology, TU Dresden, Dresden, Germany; 3 Chair of Industrial Design Engineering, TU Dresden, Dresden, Germany; 4 MySpace Lab, Department of Clinical Neuroscience, University Hospital of Lausanne, Lausanne, Switzerland; 5 Centre for Tactile Internet with Human-in-the-Loop, TU Dresden, Dresden, Germany

**Keywords:** Peripersonal space, Full-body illusion, Virtual reality, Embodiment, Multisensory integration, Aging

## Abstract

Peripersonal space (PPS) is known as the representation of the area around the body. Past research has shown that inducing a full-body illusion (FBI) can lead to PPS extending at the physical body location. However, it is unclear whether such illusions may also result in PPS transferring to a virtual body that is separate from the physical body. Furthermore, whether this effect may change over the course of natural aging is currently unknown. In three experiments, we investigated whether inducing an FBI using an avatar presented from a third-person perspective in virtual reality, results in PPS transferring to the avatar, and whether this effect differs between young (YAs; 18–29 years) and older adults (OAs; 65–83 years). We utilized a manipulation involving either synchronous or asynchronous stroking for FBI induction and assessed ratings of avatar ownership as a subjective indicator of embodiment. Meanwhile effects on PPS were measured by differences in multisensory facilitation within a visuo-tactile task. While we found significantly stronger ownership ratings for synchronous compared to asynchronous stroking in both YAs and OAs, this difference, as well as the degree of ownership, was significantly weaker for OAs. Moreover, while a distance-dependent modulation of multisensory facilitation reflecting PPS at the avatar’s location was observed in two samples of YAs, this effect was absent in OAs. These findings suggest that PPS in YAs can transfer to an avatar in virtual reality, while age-related differences in susceptibility to the FBI might contribute to a lack of this effect in OAs.

**Supplementary Information:**

The online version contains supplementary material available at 10.1007/s00221-025-07132-6.

## Introduction

The neurocognitive representation of the area directly surrounding one’s body is commonly known as peripersonal space (PPS) and plays an important role in guiding perception and action (Rizzolatti et al. 1981, 1997; Bufacchi and Iannetti 2018; Serino 2019). PPS is thought to be represented in the brain as a graded field, in which the integration of multisensory stimuli on the body (tactile) and in the environment (visual/auditory) is stronger near the body and gradually reduces with distance. This graded organization is believed to support adaptive interactions with the environment by prioritizing multisensory stimuli that are most relevant for action and defense (Bufacchi and Iannetti 2018). This is in line with results consistently observed in both monkeys and humans, showing a preferential response to multisensory stimuli close to and looming towards the body, compared to stimuli far from and receding away from the body (Fogassi et al. 1996; Graziano et al. 1997; Bufacchi and Iannetti 2018).

Interestingly, the representation of PPS has been shown to be flexible and can be influenced by a multitude of factors, including tool-use, external constraints, and social interaction (Serino 2019). These factors can lead to changes in PPS, such as tool-use resulting in a new representation of PPS forming around the end of the tool (Holmes et al. 2007; Holmes 2012), immobilizing a body part causing PPS to shrink (Bassolino et al. 2015; Toussaint et al. 2020), or social collaboration resulting either in PPS merging with that of the collaborative partner (Teneggi et al. 2013) or extending irrespective of the collaborator’s location (Hobeika et al. 2019). A more recent development in the PPS literature has been the exploration of how PPS adapts in virtual reality (VR) environments (e.g., Serino et al. 2018a, b; Fossataro et al. 2020; Petrizzo et al. 2024). Understanding how PPS adapts to VR settings can provide valuable insights for designing applications that promote plausible perception and enable successful user-interactions in VR (Lee et al. 2016; Bassano et al. 2018; Buck et al. 2020). Moreover, VR allows experimental manipulations of PPS that would not be possible in a physical setting.

An example is the third-person virtual full-body illusion (FBI; Ehrsson 2022). During the FBI, participants feel touch on their back, while viewing an avatar in VR being touched on the same location on the back. This visuo-tactile stimulation leads to participants perceiving a sense of ownership for the avatar and a shift in self-location to the avatar (Lenggenhager et al. 2007; Nakul et al. 2020). This avatar embodiment (i.e., transfer of body ownership and change in self-location), is accompanied by effects on PPS, as during the FBI, an extension of PPS from the physical body location towards the avatar’s location has been shown (Noel et al. 2015b; Salomon et al. 2017; Brizzi et al. 2023).

However, as these previous studies focused on examining the effect of the FBI on PPS relative to the physical body by only including stimuli looming towards the participant, it remains unknown whether induction of the FBI may lead to PPS emerging at the location of the virtually presented avatar. Since PPS is defined as the representation of the area around the body, and as the FBI is intended to create a shift in self-location and a sense of ownership for the avatar body, one would expect a representation of PPS emerging at the location of the avatar in VR following an FBI induction. It is important to investigate this hypothesized FBI induced transfer of PPS in VR to ensure a comprehensive understanding of the connection between avatar embodiment and PPS, as well as to clarify whether PPS can transfer to an embodied virtual location that is separate from the physical body.

An additional gap in the current literature is that most available data relate to younger individuals. Consequently, it remains unclear whether the effect of the FBI on PPS in VR may change over the course of natural aging. Exploring this could improve our understanding of how aging may influence PPS adaptation in virtual settings and inform the development of age-inclusive virtual technologies tailored to the specific perceptual and bodily experiences of older users (Li and Fitzek 2023). While the literature on age differences in the experience of the FBI in VR is still sparse, initial results hint that susceptibility to the FBI may decrease with age. An example is that middle-aged women have been shown to be less susceptible to the FBI compared to younger women (Serino et al. 2018a, b). There are more available results on the effect of age on the closely related rubber-hand illusion, in which participants are made to feel a sense of ownership for a fake rubber hand (Botvinick and Cohen 1998). However, the evidence from these studies is inconclusive, with some showing age-related differences (Marotta et al. 2018; Ferracci and Brancucci 2019), whereas others have shown comparable rubber-hand illusion effects between younger and older individuals (Campos et al. 2018, 2021). Thus, while it has been shown that PPS remains intact in old age (Sorrentino et al. 2021), if susceptibility to the FBI is affected by age, then the hypothesized FBI induced transfer of PPS to the avatar may also be affected in older adults.

To investigate the hypothesized FBI induced transfer of PPS, we induced the third-person FBI in VR as in Nakul et al. (2020) and assessed the effect on PPS with an adapted visuo-tactile task (Kuroda and Teramoto 2021, 2022; see Methods for details). The visuo-tactile task is commonly used to measure the representation of PPS by leveraging its distance and direction sensitivity. In this task, participants are required to make speeded responses to tactile stimuli, while task-irrelevant visual stimuli either loom towards or recede away from the participant (Serino et al. 2015, 2018a, b; Stone et al. 2018). The response time to the tactile stimuli varies as a function of the distance and direction of the visual stimuli, with multisensory facilitation (faster responses compared to unimodal tactile stimuli) expected to increase as the visual stimuli get closer to the participant’s body. The resulting pattern of multisensory facilitation is then used as a measure of PPS.

Crucially, in contrast to previous studies testing the effect of the FBI on PPS relative to the physical body using a visuo-tactile task with only looming stimuli (e.g. Noel et al. 2015b; Salomon et al. 2017; Brizzi et al. 2023), we wanted to focus on investigating PPS relative to the avatar by including receding stimuli. Typically there is no or a weaker distance-dependent modulation of multisensory facilitation for receding stimuli compared to looming stimuli (e.g. Serino et al. 2015; de Haan et al. 2016; Bisio et al. 2017; Kandula et al. 2017; Stone et al. 2018; Matsuda et al. 2021). Indeed, the main properties of PPS under normal conditions are a distance and direction sensitivity such that stimuli looming towards and close to the body are prioritized (Bufacchi and Iannetti 2018). However, in the context of the third-person FBI, stimuli receding away from the participant’s physical body are looming towards the avatar in the virtual space. Thus, if induction of the FBI leads to a generalization of PPS to the avatar’s location, the same distance and direction preference should emerge for receding stimuli, demonstrating that these properties are referenced to the avatar’s location.

## Overview of experiments and hypotheses

We aimed to first establish the hypothesized FBI induced transfer of PPS to the avatar in VR in young adults (Experiment 1 and 2) and then explore the potential impact of aging on this effect in older adults (Experiment 3).

In Experiment 1, we wanted to confirm the expected multisensory facilitation pattern for looming and receding visual stimuli in a sample of young adults (YAs) in a neutral no-illusion context. This gave us a neutral PPS baseline to later compare FBI effects to. In this initial experiment, instead of presenting an avatar in front of participants, we presented a neutral object that resembled the shape of an avatar and had participants complete the visuo-tactile task. In this neutral no-illusion context we expected an interaction between direction and distance. Specifically, for looming stimuli we expected a distance-dependent modulation of multisensory facilitation relative to the physical body, whereas for receding stimuli, we expected no distance-dependent modulation of multisensory facilitation.

For Experiment 2, a new sample of YAs completed the same visuo-tactile task, however, this time we induced the FBI using an avatar presented in front of participants and an intermittent stroking paradigm of either synchronous or asynchronous stroking (Nakul et al. 2020). The asynchronous stroking served as a control condition against which the expected stronger illusion effects for synchronous stroking could be compared. Due to the expected stronger illusion effects for synchronous stroking, we anticipated a stronger transfer of PPS to the avatar in this condition. This would be indicated by an interaction between stroking condition and distance, showing a stronger distance-dependent modulation of multisensory facilitation for receding stimuli close to the avatar in the synchronous compared to the asynchronous condition.

Finally, in Experiment 3, we compared the effect of the FBI on PPS between a new sample of YAs to a sample of older adults (OAs). We utilized the same setup as in Experiment 2, but due to our focus on examining a potential transfer of PPS to the avatar and informed by the results of Experiment 2 showing (as predicted) this effect specifically for receding stimuli, only receding stimuli were included in Experiment 3. In Experiment 3, we expected to replicate the interaction between stroking condition and distance observed for receding stimuli in Experiment 2 in YAs. Due to the sparse and inconclusive literature on age differences in the experience of body illusions, we did not have a clear expectation for this interaction for OAs but explored it in this sample as well.

## Methods

### Participants

Our sample size estimation was calculated using MorePower 6.0 (Campbell and Thompson 2012) for the interaction between stroking condition and distance, a power of 0.8, and an effect size of *η*^*2*^ = 0.12. The effect size was based on the reported effect of a similar interaction observed in a previous study (Noel et al. 2015b). This resulted in a suggested sample size of 20 participants. To account for potential dropouts, we recruited two samples of 25 YAs in Experiment 1 and 2 respectively. To further accommodate for the greater inter- and intraindividual variance in older adults’ reaction times (Hultsch et al. 2002; Papenberg et al. 2013), we increased the sample size in Experiment 3 by an additional 20%, resulting in a sample of 30 YAs and 30 OAs.

Across all three experiments (total *N* = 110), three participants were excluded due to a technical failure during testing, two were removed for having more than 20% false alarms in catch trials, and eight were removed due to having less than 80% visuo-tactile trials after trial filters were applied (see Statistical Analysis for trial filters). The final effective sample included in analysis was 22 YAs for Experiment 1 (16 females, mean age = 22.55 years, range = 20–28 years), 22 YAs for Experiment 2 (14 females, mean age = 22.77 years, range = 19–28 years), and 27 YAs and 26 OAs for Experiment 3 (YAs: 18 females, mean age = 22.63 years, range = 18–29 years; OAs: 11 females, mean age = 73.92 years, range = 65–83 years).

All participants were right-handed, had normal or corrected-to-normal vision, reported normal tactile perception, and had no history of neurological or current psychiatric disorders. Participation in the study was compensated with 10€ per hour, or course credit for students who requested and preferred this. The study was approved by the ethics committee of the TU Dresden (SR-EK-5012021) and was conducted in accordance with the 1964 Declaration of Helsinki. All participants provided written informed consent prior to participation.

### Apparatus

The visual stimuli and virtual scene were implemented in Unity 3D (Unity Technologies, United States) using the Unity Experiment Framework (Brookes et al. 2020) and displayed on an HTC VIVE VR headset (Valve Corporation, United States). Tactile stimuli were presented using a vibrotactile stimulator (Vibrating Mini Motor Disc #1201, Adafruit, USA) that was controlled by an ESP32-PICO-D4 microcontroller (Espressif Systems, China). A response button was attached to the same microcontroller to record speeded responses to the tactile stimuli.

To address known display latency in VR headsets, we used the photodiode method from Le Chénéchal and Chatel-Goldman (2018) to compare the VR headset’s display latency with the microcontroller’s execution latency for tactile stimuli. The headset showed a mean latency of 36 ms (*SD* < 1 ms), while the microcontroller’s latency was under 1 ms (*SD* < 0.1 ms). To synchronize visual and tactile stimuli, a 36 ms delay was added to tactile stimulus delivery by the microcontroller.

**Fig. 1:**
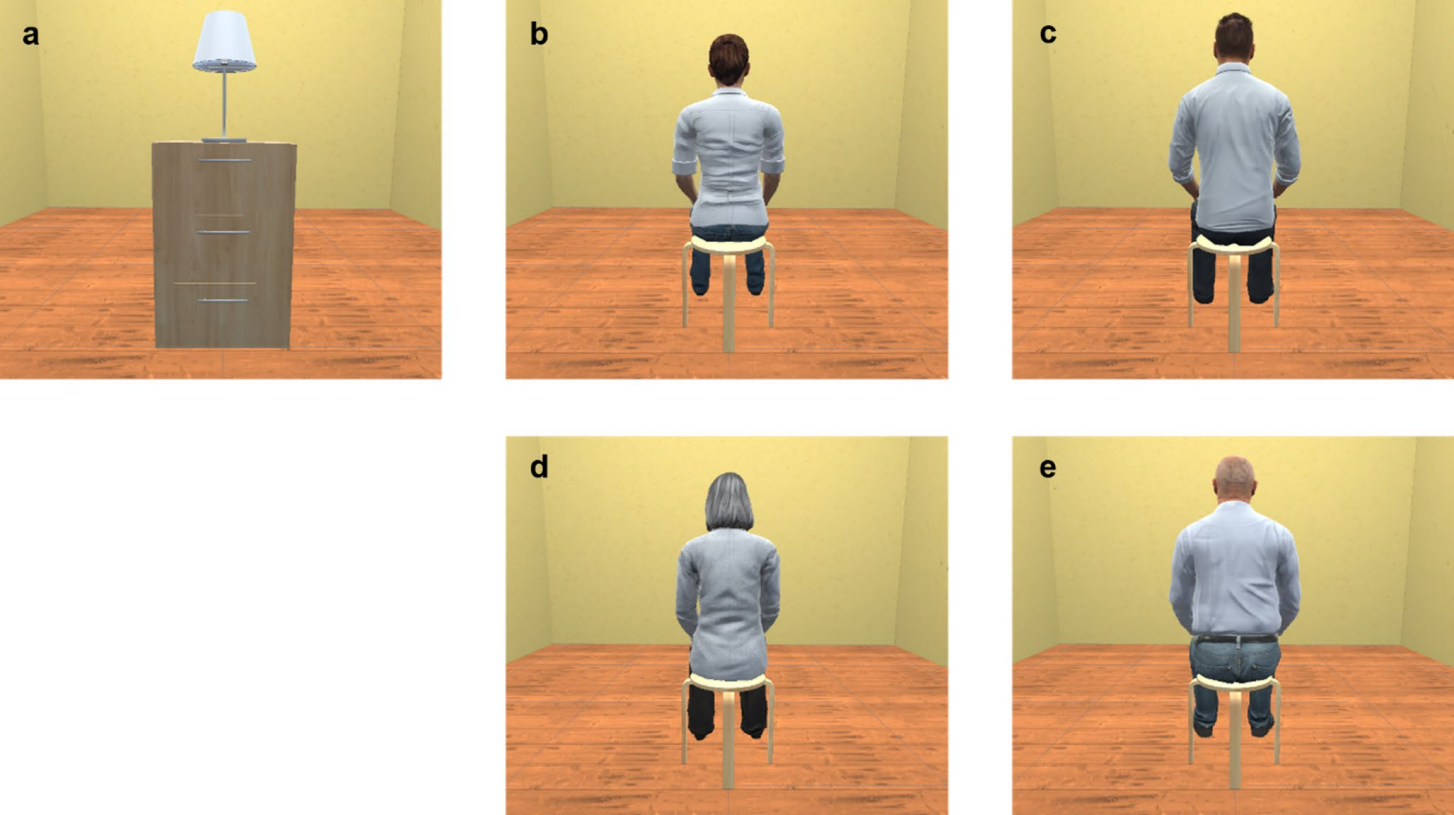
The virtual scene as presented in each experiment. (**a**) The neutral object used for testing under a no-illusion context in Experiment 1. The female (**b**) and male (**c**) avatar used for illusion induction for YAs in Experiment 2 and 3. The female (**d**) and male (**e**) avatar used for illusion induction for OAs in Experiment 3. The scenes were presented 2 m in front of participants on an HTC VIVE VR headset

The virtual room used in the setup matched the size (5 m × 7 m), floor, and wall color of the physical room in which the experiment took place. In Experiment 1, a neutral object, consisting of a drawer with a lamp (mimicking the shape of the avatars), was presented 2 m in front of participants’ viewpoint. The use of an object instead of passively presenting an avatar from a third-person view, was to ensure Experiment 1 represented a neutral no-illusion context, as passively viewing a body from a third-person perspective has been shown to affect tactile perception, whereas viewing an object does not have this effect (Aspell et al. 2009). In Experiment 2 and 3 the neutral object was replaced with an age and gender matched avatar sitting on a stool (see Fig. 1). The avatars were taken from the Microsoft Rocketbox library (Gonzalez-Franco et al. 2020) and lightly modified. At block initialization, the neutral object or avatar was automatically scaled to the participant’s height using the VR headset data. The height of visual stimuli was set based on the same principle. To minimize visual distractions the rest of the virtual room was empty.

### Avatar ownership ratings

Participants’ responses to the standardized Embodiment Questionnaire (Peck and Gonzalez-Franco 2021) were used to assess differences in subjective effects of the FBI between the synchronous and asynchronous condition. Responses were given on a 7-point Likert scale ranging from “1 = strongly disagree” to “7 = strongly agree”. We focused on the ownership subscale of the questionnaire and calculated the ownership score as suggested in Peck and Gonzalez-Franco (2021; see text in Supplementary Materials for calculation). The questionnaire was translated into German and independently verified by two native German speakers. The English version of the questionnaire can be found in Table S1 and the translated German version in Table S2 in the Supplementary Materials.

### Visuo-tactile task for assessing PPS

A visuo-tactile task was used to assess the effects of the FBI on PPS. Participants were required to make speeded responses to tactile stimuli presented simultaneously at sternum level on the front and back of the trunk (Experiment 1 and 2), or just at sternum level on the back of the trunk (Experiment 3), during the presence or absence of concurrent task-irrelevant visual stimuli (green ball of 8 cm diameter). All trials began with the presentation of a red fixation cross for 1000 ms. This was followed by a random delay between 1000 ms and 1500 ms before continuing with one of the three possible trial types. Visuo-tactile trials continued with presentation of the visual stimulus for 300 ms plus a random jitter (ranging between − 30 ms and + 30 ms in 1 ms steps), followed by 100 ms of simultaneous visual and tactile stimulus presentation. Participants then had 1000 ms to respond. Unimodal tactile trials followed the same timing as visuo-tactile trials but without adding a jitter or the visual stimulus being presented. Catch trials also followed the same timing, but without adding a jitter or the tactile stimulus being presented. Since catch trials did not include a tactile stimulus, no response was required and responses given were considered false alarms to the visual stimulus. For this reason, the 1000 ms response window began from onset of the visual stimulus in catch trials.

For trials including a visual stimulus, the visual stimulus would appear on the left or right of the visual midline (counterbalanced across trials), along a 5.7° diagonal line. To assist differentiation of the visual stimulus movement direction, as well as to make its predicted point of impact clearer, the diagonal line pointed towards the participant for looming trials and towards the avatar for receding trials. Once appeared, the visual stimulus would loom towards or recede away from the participant’s viewpoint at a speed of 33.5 cm/s for 300 ms (plus random jitter in visuo-tactile trials) along the diagonal, until it reached one of the six possible distances from the participant (0.3 m, 0.6 m, 0.9 m, 1.2 m, 1.5 m, 1.8 m; between − 0.01 m and + 0.01 m offset depending on jitter in visuo-tactile trials). The visual stimulus would then continue moving for another 100 ms simultaneously to presentation of the tactile stimulus in visuo-tactile trials, or on its own in catch trials.

This mini loom/recede design, in which (regardless of distance level) visual stimuli moved for the same short duration before tactile stimulus presentation (Kuroda and Teramoto 2021, 2022), is a deviation from previous experiments utilizing this task (e.g., Serino et al. 2015, 2018a, b; Stone et al. 2018). In these previous experiments, visual stimuli would move across the entire tested area, with the tactile stimulus being administered after varying temporal delays. The mini loom/recede design is an important alteration to this, as it ensures the same temporal expectancy for each distance level and therefore addresses concerns about the differentiation of spatial from temporal expectancy effects (Kandula et al. 2017).

**Fig. 2:**
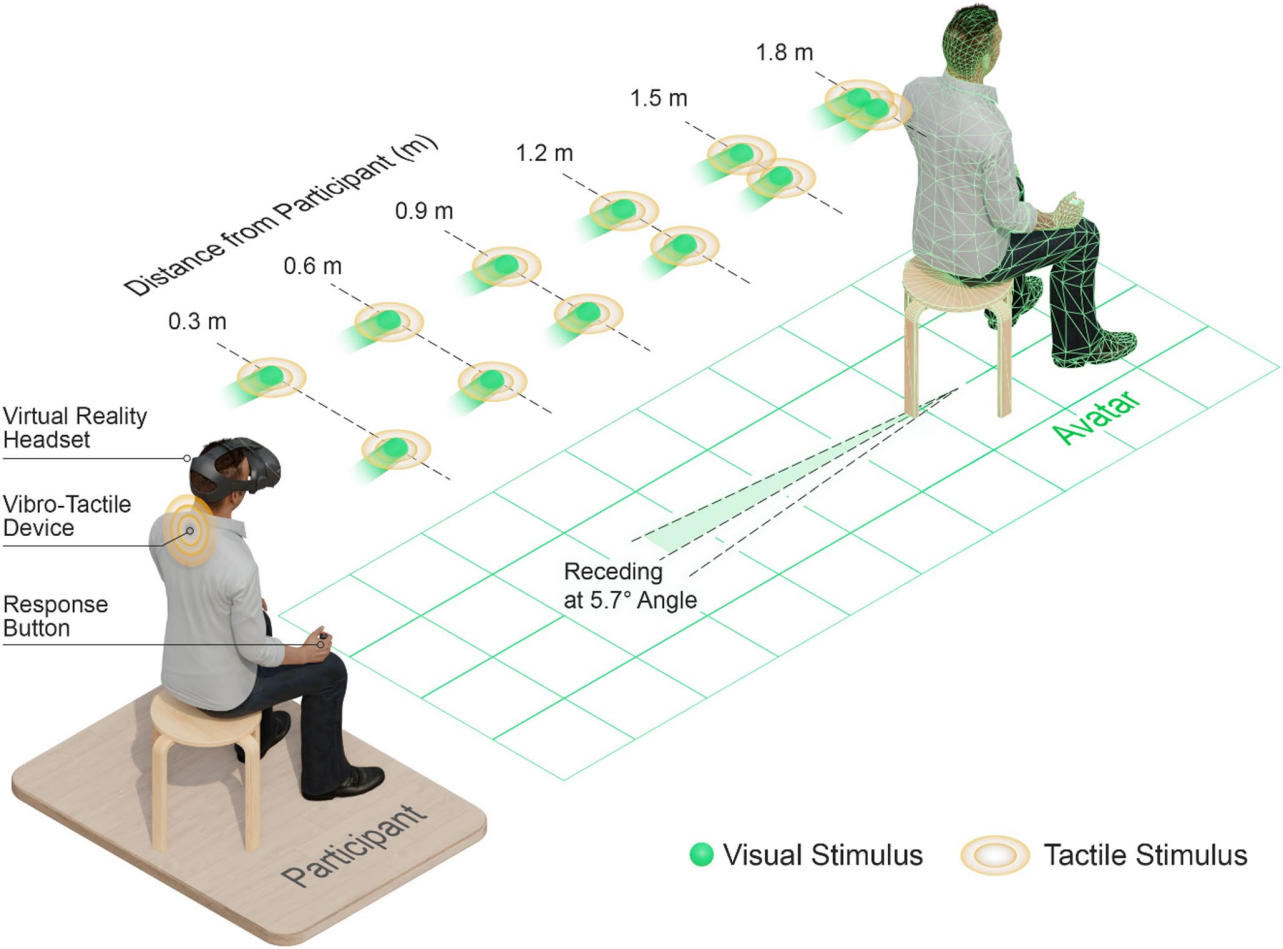
Illustration of the visuo-tactile task for measuring PPS based on the version used in Experiment 3. Participants were required to make speeded responses to tactile stimuli presented at sternum level on the back of the trunk during the presence of task-irrelevant visual stimuli (green ball) shown on a VR headset. The visual stimulus appeared along a (left- or right-sided) 5.7° diagonal and receded towards one of six possible distance levels between the participant and an avatar shown 2 m in front in the virtual scene. Critically, the visual stimulus appeared shortly before the distance levels and thus moved for the same duration regardless of distance level. Once the visual stimulus reached the distance level, the tactile stimulus was presented, and participants were required to press the response button. In addition to these visuo-tactile trials, there were unimodal tactile trials in which only the tactile stimulus was presented and catch trials in which only the visual stimulus was presented. Since no tactile stimulus was presented in catch trials, no response was required in these trials. Importantly, Experiment 3 included only receding trials, whereas Experiment 1 and 2 included receding and looming trials (visual stimuli loomed towards the participant). Thus, in Experiment 1 and 2 the 5.7° diagonal for visual stimuli pointed towards the participant instead of the avatar in looming trials (but with the same distance levels) and tactile stimuli were presented simultaneously at sternum level on the front and back of the trunk

The choice to include only receding stimuli in Experiment 3 was motivated by our focus on examining a potential transfer of PPS to the avatar’s location and the results from Experiment 2 showing that this effect can be seen specifically for receding stimuli. This had the additional benefit that we could increase the number of trials per factor level to counteract the greater inter- and intraindividual reaction time variance in older adults (Hultsch et al. 2002; Papenberg et al. 2013), while also keeping the experiment at a manageable length for older adults (roughly 2 h). A visualization of the visuo-tactile task, as used in Experiment 3, is shown in Fig. 2.

### Procedure

After completing a practice block of the visuo-tactile PPS task, participants proceeded to the experimental blocks. In Experiment 2 and 3 each experimental block began with a one-minute illusion elicitation period. This consisted of tactile stroking with a VR controller on the participant’s back, in addition to visual stroking with a virtual model of the VR controller on the back of the avatar in the virtual scene (see Nakul et al. 2020). In the synchronous condition the tactile and visual stroking were presented in synchrony, whereas in the asynchronous condition the visual stroking followed the tactile stroking with a 500 ms delay. The initial one minute of stroking was followed by five trials of the visuo-tactile PPS task, which in turn was followed by another 10 s of stroking. This procedure of five PPS trials followed by 10 s of stroking continued throughout the block to prevent the illusion effects from waning. At the end of each block participants filled out the Embodiment Questionnaire (Peck and Gonzalez-Franco 2021).

As Experiment 1 represented a neutral no-illusion context, PPS trials were presented without intermittent stroking, and participants did not respond to the Embodiment Questionnaire. Thus, Experiment 1 consisted of two blocks of just the visuo-tactile task. On the other hand, as Experiment 2 and 3 involved intermittent stroking, we used within-subject reverse counterbalancing in a fixed order for the two stroking conditions (synchronous vs. asynchronous) across four experimental blocks (i.e., AB-BA). Thus, each participant experienced the synchronous condition (A) once before the asynchronous condition (B) and then also once in the reversed order.

In Experiment 1 and 2 each block consisted of 36 looming visuo-tactile trials (six per distance level), 36 receding visuo-tactile trials (six per distance level), 24 unimodal tactile trials, 12 looming catch trials (two per distance level), and 12 receding catch trials (two per distance level). Based on results from Experiment 2, Experiment 3 focused only on receding stimuli with blocks including 60 receding visuo-tactile trials (10 per distance level), 21 unimodal tactile trials, and 24 receding catch trials (four per distance level). Across all three experiments, the order of visuo-tactile, unimodal tactile, and catch trials was randomized within blocks.

### Statistical analysis

Trials with extremely fast (< 100 ms) or slow (> 1000 ms) reaction times were removed from analysis. Additionally, in Experiment 1 and 2, unimodal tactile trials with reaction times ± 3 SDs from the block average and visuo-tactile trials with reaction times ± 3 SDs from the block and visual direction average were removed as outliers. This resulted in the rejection of 1.6% of trials in Experiment 1 and 2.5% in Experiment 2. Since there were more visuo-tactile trials per stroking condition and distance level in Experiment 3 (20 vs. 12 in Experiment 1 and 2), outlier analysis in this experiment could be done at an individual-level to account for greater inter- and intraindividual variance in older adults’ reaction times (Hultsch et al. 2002; Papenberg et al. 2013). Here, unimodal tactile and visuo-tactile trials with reaction times ± 3 SDs from each participant’s block average were removed, resulting in the rejection of 3.2% of trials for YAs and 7.2% for OAs.

Following these trial filters, only participants who had at least 80% visuo-tactile trials remaining were included in analysis. Additionally, only participants who had less than 20% false alarms for catch trials were included. The remaining participants constituting the final effective sample for analyses all performed at a high level, with a mean percentage of false alarms in catch trials of 3.4% in Experiment 1, 3.5% in Experiment 2, 0.7% in YAs in Experiment 3, and 1.4% in OAs in Experiment 3.

To test the hypothesized effects on PPS in all experiments, the multisensory reaction time facilitation (RTF) when responding to tactile stimuli (at sternum level on the back and front of the torso in Experiment 1 and 2 and only on the back of the torso in Experiment 3) during simultaneous presentation of task-irrelevant visual stimuli was used as the dependent variable. RTF was calculated by subtracting reaction times in unimodal tactile trials from those in visuo-tactile trials (Noel et al. 2015a; Serino et al. 2015; Salomon et al. 2017). Thus, a negative RTF signifies multisensory facilitation with quicker reaction times in visuo-tactile compared to unimodal tactile trials. The mean RTF values were calculated for each participant individually and for each level of the within-subject factors included in the three experiments.

All data analysis was done in R Statistical Software (v4.3.1; R Core Team 2023). The assumption of sphericity for within-subject ANOVA factors was tested using Mauchly’s test and violations of sphericity were corrected using the Greenhouse-Geisser correction. For between-subject ANOVA factors, the homogeneity of variance was tested using Levene’s test (all *p* >.05). The normality of ANOVA model residuals was assessed using QQ-plots and frequency histograms. Where appropriate, post-hoc t-tests (or Wilcoxon tests in the case of non-normal data) were corrected for multiple testing using the Holm-Bonferroni method. P-values corrected using this method are marked with the subscript *adjusted* in the Results section. All post-hoc tests were conducted as two-tailed tests.

## Results

### Experiment 1: Looming and receding results in neutral no-illusion context

**Fig. 3:**
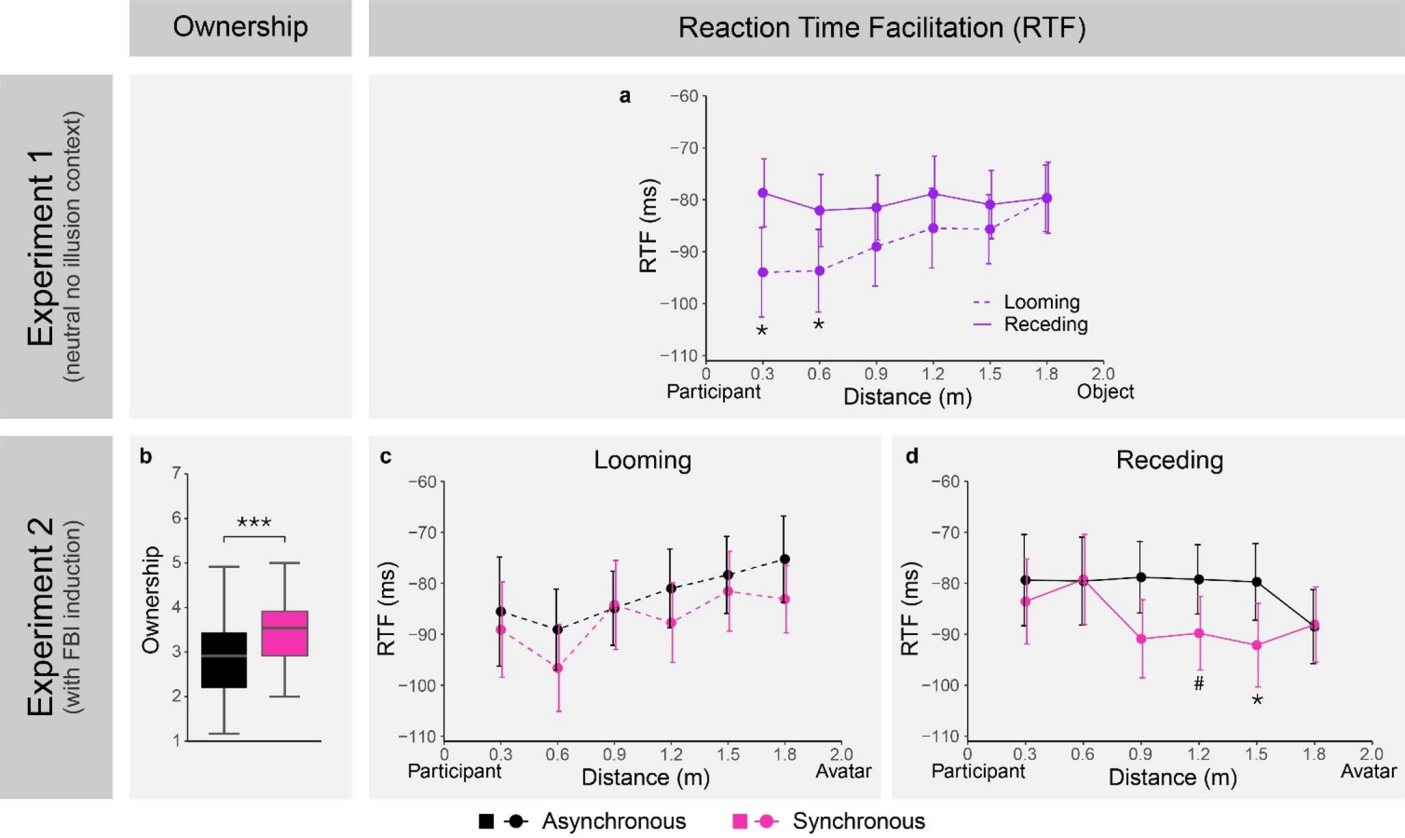
Plots of RTF results from Experiment 1 (neutral no-illusion context) and 2 (with FBI induction) by key experimental factors, as well as ownership scores in Experiment 2. (**a**) RTF as a function of direction and distance in Experiment 1. (**b**) Box and whisker plot showing ownership scores by stroking condition in Experiment 2. RTF as a function of stroking condition and distance for looming (**c**) and receding trials (**d**) in Experiment 2. For the RTF plots, negative RTF signifies a quicker reaction time in visuo-tactile compared to unimodal tactile trials, error bars show ± 1 standard error of the mean, and the location of the participant and avatar/neutral object are shown on each end of the x-axis. For the box and whisker plot, the line in the middle of the box shows the median, the box represents the first and third quartile, and the whiskers represent the lowest value within 1.5 × IQR below the first quartile and the highest value within 1.5 × IQR above the third quartile. An asterisk represents a significant difference between directions in plot (**a**), whereas in plots (**b-d**) an asterisk represents a significant difference between stroking conditions. The hashtag in plot (**d**) represents a marginally significant (*p*_*adjusted*_ =.051) difference between stroking conditions

The mean RTF in Experiment 1 was analyzed by a two-way ANOVA with the within-subject factors direction (two levels: looming, receding) and distance (six levels: 0.3 m, 0.6 m, 0.9 m, 1.2 m, 1.5 m, 1.8 m). This revealed a significant main effect of direction (*F*(1, 21) = 12.27, *p* =.002, *η*_*p*_^*2*^ = 0.37), a significant main effect of distance (*F*(5, 105) = 2.95, *p* =.016, *η*_*p*_^*2*^ = 0.12), and a significant interaction between direction and distance (*F*(5, 105) = 2.32, *p* =.048, *η*_*p*_^*2*^ = 0.10). To further understand this interaction (see Fig. 3a), we analyzed the simple main effect of distance on RTF separated by direction. While there was a significant main effect of distance for looming stimuli (*F*(5, 105) = 5.03, *p* <.001, *η*_*p*_^*2*^ = 0.19), there was no main effect of distance for receding stimuli (*F*(5, 105) = 0.33, *p* =.892, *η*_*p*_^*2*^ = 0.02). Additionally, post-hoc comparisons between looming and receding stimuli for each distance level revealed that RTF was significantly greater for looming compared to receding stimuli at 0.3 m (*V* = 215, *p*_*adjusted*_ =.014, *r* =.61), as well as at 0.6 m (*t*(21) = 3.78, *p*_*adjusted*_ =.007, *d* = 0.81), while this comparison was not significant at the other distance levels (*p*_*adjusted*_ >.05).

Taken together, the results for Experiment 1 show that in a neutral no-illusion context, there is a distance-dependent modulation of multisensory facilitation for looming stimuli, with greater multisensory facilitation close to the physical body, whereas there is no distance-dependent modulation of multisensory facilitation for receding stimuli. This is in line with findings showing that PPS around the physical body responds preferably to looming compared to receding stimuli (Fogassi et al. 1996; Graziano et al. 1997; Bufacchi and Iannetti 2018) and there usually being no (or a weaker) distance-dependent modulation of multisensory facilitation for receding stimuli (e.g., Serino et al. 2015; de Haan et al. 2016; Kandula et al. 2017).

### Experiment 2: Looming and receding results under induction of FBI

As a manipulation check, ownership scores between the synchronous and asynchronous stroking condition were compared using a Wilcoxon signed-rank test. This revealed significantly greater ownership ratings in the synchronous compared to the asynchronous condition (*V* = 219, *p* <.001, *r* =.78), suggesting that synchronous stroking successfully elicited a stronger FBI effect compared to asynchronous stroking (see Fig. 3b).

A three-way ANOVA with the within-subject factors direction (two levels: looming, receding), stroking condition (two levels: synchronous, asynchronous), and distance (six levels: 0.3 m, 0.6 m, 0.9 m, 1.2 m, 1.5 m, 1.8 m) was used to analyze the mean RTF. This revealed a significant main effect of stroking condition (*F*(1, 21) = 4.76, *p* =.041, *η*_*p*_^*2*^ = 0.19), a significant two-way interaction between direction and distance (*F*(5, 105) = 8.81, *p* <.0001, *η*_*p*_^*2*^ = 0.30), and a significant three-way interaction between direction, stroking condition, and distance (*F*(5, 105) = 2.93, *p* =.016, *η*_*p*_^*2*^ = 0.12).

To investigate the three-way interaction further, we conducted additional two-way ANOVAs on RTF split by direction with the factors stroking condition and distance. For looming stimuli we found a significant main effect of stroking condition (*F*(1, 21) = 4.69, *p* =.042, *η*_*p*_^*2*^ = 0.18) and a significant main effect of distance (*F*(3.27, 68.71) = 4.04, *p* =.009, *η*_*p*_^*2*^ = 0.16), but no interaction between stroking condition and distance (*F*(5, 105) = 0.79, *p* =.558, *η*_*p*_^*2*^ = 0.04). This signifies a distance-dependent modulation of multisensory facilitation relative to the physical body (as in Experiment 1) and a difference in multisensory facilitation between stroking conditions, showing stronger multisensory facilitation in the synchronous than asynchronous condition; however, these effects were independent of each other for looming stimuli, such that the distance-dependent modulation of multisensory facilitation did not differ between stroking conditions (see Fig. 3c).

On the other hand, the two-way ANOVA for receding stimuli revealed a significant interaction between stroking condition and distance (*F*(5, 105) = 2.64, *p* =.027, *η*_*p*_^*2*^ = 0.11), which shows that the distance-dependent modulation of multisensory facilitation differed between stroking conditions. To better understand this interaction for receding stimuli we analyzed the simple main effect of distance on RTF in both stroking conditions. This revealed a significant main effect of distance in the synchronous (*F*(5, 105) = 2.98, *p* =.015, *η*_*p*_^*2*^ = 0.12), but not in the asynchronous condition (*F*(3.31, 69.43) = 1.62, *p* =.189, *η*_*p*_^*2*^ = 0.07). Post-hoc comparisons at each distance level between synchronous and asynchronous stroking found a significantly greater RTF for synchronous stroking at 1.5 m (*t*(21) = 3.82, *p*_*adjusted*_ =.006, *d* = 0.82) and a marginally significant difference at 1.2 m (*t*(21) = 2.82, *p*_*adjusted*_ =.051, *d* = 0.60). No significant differences were found at the other distance levels (*p*_*adjusted*_ >.05). These results (see Fig. 3d) suggest that the FBI, elicited in the synchronous condition, resulted in a distance-dependent modulation of multisensory facilitation for receding stimuli in proximity to the avatar.

Taken together, the results of Experiment 2 imply that induction of the FBI leads to a transfer of PPS to the avatar.

### Experiment 3: Receding results under induction of FBI between YAs and OAs

**Fig. 4:**
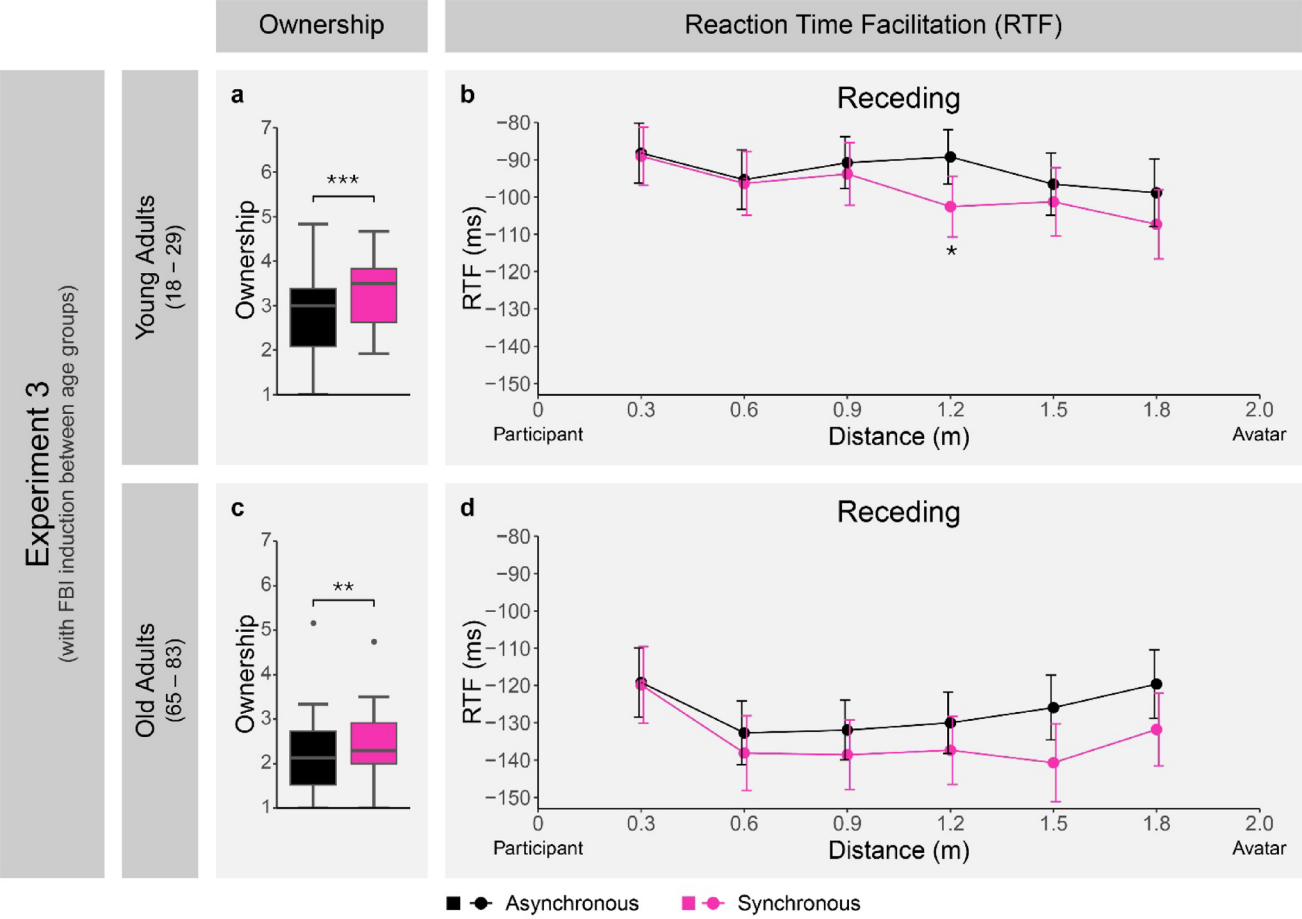
RTF and ownership score results for YAs (top panel) and OAs (bottom panel) in Experiment 3. Box and whisker plot showing ownership scores by stroking condition for YAs (**a**) and OAs (**c**). RTF as a function of stroking condition and distance for YAs (**b**) and OAs (**d**). For the RTF plots, negative RTF signifies a quicker reaction time in visuo-tactile compared to unimodal tactile trials, error bars show ± 1 standard error of the mean, and the location of the participant and avatar are shown on each end of the x-axis. For the box and whisker plots, the line in the middle of the box shows the median, the box represents the first and third quartile, and the whiskers represent the lowest value within 1.5 × IQR below the first quartile and the highest value within 1.5 × IQR above the third quartile. Following standard box-plot conventions, values more than either 1.5 × IQR below the first quartile or 1.5 × IQR above the third quartile are visualized as grey dots. Importantly, the two values shown exceeding this threshold in plot (**c**) were not removed from analysis. Across all plots, a significant difference between stroking conditions is represented with an asterisk

Separate Wilcoxon signed-rank tests (due to comparing paired Likert scale data) for both YAs and OAs were used to compare ownership scores between the synchronous and asynchronous condition. This revealed significantly greater ownership ratings for synchronous compared to asynchronous stroking for both YAs (*V* = 329.5, *p* <.0001, *r* =.75; see Fig. 4a) and OAs (*V* = 213, *p* =.005, *r* =.57; see Fig. 4c). However, Wilcoxon rank-sum tests (due to comparing independent Likert scale data) revealed that ownership ratings in the synchronous condition were significantly lower for OAs compared to YAs (*W* = 559.5, *p* <.001, *r* =.51) and that the difference in ownership ratings between synchronous and asynchronous stroking (calculated as synchronous scores - asynchronous scores) was significantly smaller for OAs than for YAs (*W* = 496, *p* =.010, *r* =.36). This indicates that we were successful in creating stronger FBI effects in the synchronous compared to the asynchronous condition for both YAs and OAs, but that this effect was weaker and not as differentiated for OAs.

Note that when visualizing the ownership results, to follow standard box-plot conventions, ownership scores falling more than 1.5 × IQR below the first quartile or above the third quartile were visualized as individual grey dots. One OA participant had an ownership score exceeding this threshold in both the synchronous (2.00 × IQR above third quartile) and asynchronous (2.02 × IQR above third quartile) condition (see Fig. 4c). However, given that large inter-individual variability is expected for subjective ratings, these values were only marked for visualization purposes but were not removed from analysis.

For the analysis of mean RTF in Experiment 3, we conducted a three-way ANOVA with the between-subject factor age (two levels: young, old), and the within-subject factors stroking condition (two levels: synchronous, asynchronous) and distance (six levels: 0.3 m, 0.6 m, 0.9 m, 1.2 m, 1.5 m, 1.8 m). We found a significant main effect of age (*F*(1, 51) = 10.22, *p* =.002, *η*_*p*_^*2*^ = 0.17), a significant main effect of distance (*F*(3.56, 181.48) = 7.59, *p* <.0001, *η*_*p*_^*2*^ = 0.13), and a significant interaction between age and distance (*F*(3.56, 181.48) = 4.88, *p* =.001, *η*_*p*_^*2*^ = 0.09). The three-way interaction between age, stroking condition, and distance was however not significant (*F*(4.10, 208.99) = 0.79, *p* =.534, *η*_*p*_^*2*^ = 0.02). Note that to control for potential factors that could influence the age comparison in our setup, we conducted a covariate analysis with measured tactile thresholds, as these are known to increase with age (Verrillo 1980; Ekman et al. 2021), as well as the time spent in VR and gaming environments. As adding these factors as covariates did not affect the main patterns and interpretations of results, we here reported the ANOVA results without these covariates (for further details and results of the covariate analyses see text, Table S3 (gaming and VR questionnaire in German), and Table S4 (gaming and VR questionnaire translated into English) in the Supplementary Materials).

Despite the three-way interaction not being significant, we proceeded to conduct additional two-way ANOVAs on RTF with the within-subject factors stroking condition and distance separately for YAs and OAs. This was decided due to the exploratory nature of the factor age in our analysis. For OAs we found a significant main effect of distance (*F*(3.24, 80.97) = 5.41, *p* =.001, *η*_*p*_^*2*^ = 0.18), but no main effect of stroking condition (*F*(1, 25) = 0.89, *p* =.355, *η*_*p*_^*2*^ = 0.03), or interaction between distance and stroking condition (*F*(3.39, 84.64) = 0.95, *p* =.428, *η*_*p*_^*2*^ = 0.04). To reaffirm the absence of an interaction between stroking condition and distance in OAs and to allow a comparison to the results for YAs, we ran post-hoc comparisons of RTF between stroking conditions at each distance level and found no significant results (*p*_*adjusted*_ >.05). These results (see Fig. 4d) show that in OAs there was no FBI induced distance-dependent modulation of multisensory facilitation for receding stimuli, suggesting that there was no transfer of PPS to the avatar in this age group.

Conversely, the same analysis in YAs revealed a significant main effect of distance (*F*(3.23, 84.02) = 7.91, *p* <.0001, *η*_*p*_^*2*^ = 0.23) and a significant interaction between stroking condition and distance (*F*(5, 130) = 2.37, *p* =.043, *η*_*p*_^*2*^ = 0.08). To further investigate this interaction, we analyzed the simple main effect of distance on RTF in both stroking conditions. This revealed a significant main effect of distance in the synchronous (*F*(3.67, 95.47) = 6.72, *p* <.001, *η*_*p*_^*2*^ = 0.21) and asynchronous condition (*F*(5, 130) = 3.85, *p* =.003, *η*_*p*_^*2*^ = 0.13). Post-hoc comparisons of the RTF at each distance level between stroking conditions showed a significantly greater RTF for synchronous compared to asynchronous stroking at 1.2 m (*t*(26) = 2.90, *p*_*adjusted*_ =.045, *d* = 0.56), while the other distance levels did not show a significant difference (*p*_*adjusted*_ >.05).

Given that a simple main effect of distance was found in both stroking conditions, we conducted an additional analysis to examine the difference in strength of the distance effect between stroking conditions in YAs. For this we fit simple linear models of RTF across distance for each YA in both stroking conditions. The resulting individual model slopes in each stroking condition were then compared, showing significantly steeper negative slopes in the synchronous (*M* = − 10.93, *SD* = 12.00) compared to the asynchronous (*M* = -5.26, *SD* = 9.83) condition (*t*(26) = 2.71, *p* =.012, *d* = 0.52). This indicates a stronger increase in multisensory facilitation with increasing proximity to the avatar for synchronous compared to asynchronous stroking.

The pattern of results for YAs (see Fig. 4b) shows that the FBI led to a significant increase in multisensory facilitation for receding stimuli in proximity to the avatar, thus replicating the distance-dependent modulation of multisensory facilitation for receding stimuli observed in Experiment 2.

Taken together, the results of Experiment 3 replicate the implied transfer of PPS to the avatar under induction of the FBI observed in Experiment 2. However, this effect was only found in YAs and was not present in OAs. 

## Discussion

In a series of three experiments, we aimed to first establish whether eliciting an FBI on an avatar presented from a third-person perspective in VR would result in a transfer of PPS to the location of the avatar. Building on this, we then explored whether aging might affect this FBI induced transfer of PPS, given the inconclusive previous literature on how aging may affect susceptibility to the FBI. We first confirmed the expected multisensory facilitation pattern for looming and receding stimuli in a sample of YAs in a neutral no-illusion context (Experiment 1). We then observed an FBI induced transfer of PPS to the avatar in a new sample of YAs, through a distance-dependent modulation of multisensory facilitation as receding stimuli approached the avatar (Experiment 2). Finally, while we were able to replicate this effect in another sample of YAs, we found weaker subjective ownership ratings alongside an absence of a PPS transfer effect in a sample of OAs (Experiment 3). Together, these results suggest that PPS in young adults can transfer to an embodied location in VR that is separate from the physical body, but that reduced susceptibility to the FBI may be a factor influencing the absence of this effect in older adults.

While a previous study has demonstrated a transfer of PPS to a disconnected virtual hand in VR (Mine and Yokosawa 2021), our findings show that PPS can transfer to an entire virtual body. Importantly, this earlier study included a reaching task in which the virtual hand was used to reach towards a cylinder. This leaves open whether the observed effects were due to the virtual hand being used as a tool, similar to documented tool-use effects on PPS (e.g., Holmes et al. 2007; Holmes 2012), or whether this was related to embodiment of the virtual hand. In contrast, as our setup involved an entire avatar body and no manipulation of the avatar that could be associated with tool-use, our findings relate more closely to avatar embodiment. It would be of interest to explore the PPS effects observed for the disconnected virtual hand alongside the perceived sense of embodiment for the virtual hand. This could help clarify whether the transfer of PPS to a virtual body part and to a full virtual body may share common mechanisms.

It should be noted that the significant differences in RTF between stroking conditions for receding stimuli in YAs in both Experiment 2 and 3, were observed at distances closer to the avatar than to the participant, but not at the closest distance to the avatar (1.8 m). This may suggest that although synchronous stroking yielded larger effects than asynchronous stroking, a transfer of PPS may have also occurred in the asynchronous condition, but to a lesser extent than in the synchronous condition. This may explain the apparent increase in multisensory facilitation at 1.8 m in the asynchronous condition for receding stimuli in Experiment 2 (see Fig. 3d), as well as the simple main effect of distance observed also in the asynchronous condition for YAs in Experiment 3. Crucially, however, additional slope analysis in Experiment 3 confirmed that the effect of distance on multisensory facilitation was stronger in the synchronous condition. Nevertheless, future studies may need to include a baseline no-illusion experiment (as in Experiment 1) or a baseline no-illusion condition as a third level of the experimental manipulation, to provide a neutral comparison.

While we included a baseline no-illusion experiment, our study did not incorporate a control condition comparing the effects of synchronous versus asynchronous stroking of a neutral object for which ownership cannot arise. This limits the ability to more directly relate the observed transfer of PPS with changes in ownership. However, prior research offers important context. Aspell et al. (2009) found that synchronous (but not asynchronous) stroking alters the spatial representation of visuo-tactile stimuli when applied to a body-shaped stimulus capable of inducing the FBI, but not when applied to a neutral object. Additionally, Salomon et al. (2017) found that visuo-tactile stimulation can influence both avatar ownership and PPS, even when the stimulation is not consciously perceived. These findings support the notion that the effects of visuo-tactile stroking on PPS are linked with changes in ownership. This also aligns with a prominent theoretical perspective (Blanke 2012; Blanke et al. 2015; Grivaz et al. 2017; Ehrsson 2020), which proposes that synchronous visuo-tactile stimulation recalibrates the visuo-tactile receptive fields of PPS and this process also being a key neurophysiological mechanism underlying alterations in body ownership. Notwithstanding, future studies should consider including a control condition involving stroking of a neutral object to more directly link the transfer of PPS with changes in ownership.

Despite our focus on receding stimuli, the results for looming stimuli in Experiment 2 are noteworthy, as they relate to previous studies showing an extension of PPS relative to the physical body (Noel et al. 2015b; Salomon et al. 2017; Brizzi et al. 2023). While we observed main effects of distance and stroking condition, there was no interaction between these factors for looming stimuli. This suggests that while there was greater multisensory facilitation in the synchronous condition, the effect of distance did not differ between stroking conditions for looming stimuli. These results differ from the above-mentioned earlier studies showing an extension of PPS relative to the physical body. Methodological differences in the visuo-tactile task employed between our and prior studies likely contributed to this discrepancy. Notably, our randomized presentation of both looming and receding stimuli might have reduced the saliency of looming stimuli, compared to exclusively presenting looming stimuli. Furthermore, the mini loom/recede design we utilized instead of presenting the visual stimuli along the entire tested area, may have influenced our results by removing the effect of temporal expectancy (Kandula et al. 2017; Kuroda and Teramoto 2021, 2022). Future studies directly comparing these versions of the visuo-tactile task are needed, to better isolate the core mechanisms underlying PPS and ensure interpretations across studies are accurate and comparable.

A transfer of PPS to the avatar was not observed in OAs. This could be associated with the weaker FBI effects for OAs, as shown by their ownership ratings in Experiment 3. This is in line with the finding that middle-aged women are less susceptible to the FBI compared to younger women (Serino et al. 2018a, b). Our results therefore offer an addition to the sparse literature on age differences in the FBI and suggest that aging attenuates susceptibility to the FBI. A potential explanation for reduced susceptibility to the FBI in older adults, is that older adults are thought to be less embodied than younger adults, due to age-related neuronal degradation in the sensorimotor system (Costello and Bloesch 2017; Kuehn et al. 2018). It could be that the same age-related neuronal degradation that affects general embodiment, also affects the ability to experience illusions such as the FBI. Future studies should continue to investigate what may cause differences in subjective experience of the FBI between young and older adults.

Note that although controlling for individual differences in time spent gaming did not change the pattern of our results (see covariate analysis text in Supplementary Materials), young adults might still be more accustomed to a third-person perspective due to contact with digital media. As it has been shown that embodiment of virtual avatars is stronger from a first-person than a third-person perspective (Debarba et al. 2017), it would be of value to test whether older adults experience stronger illusion effects comparable to that of young adults during a first-person based FBI induction in VR, when compared to the third-person FBI. This would help in understanding whether older adults are generally less susceptible to body illusions in VR, or if they have specific difficulties adapting to the third-person perspective.

An interesting observation is that despite OAs not showing a transfer of PPS to the avatar, they did show greater (i.e., more negative) overall RTF compared to YAs (see Fig. 4b vs. Figure 4d). This is in line with older adults showing reduced unisensory tactile sensitivity (Verrillo 1980; Ekman et al. 2021), which in turn can contribute to stronger multisensory facilitation effects through concurrent visual stimuli, as predicted by the principle of inverse effectiveness (Stein and Stanford 2008).

A few limitations should be kept in mind when considering the here observed age differences. We did not observe a significant three-way interaction between age, stroking condition, and distance. The absence of this three-way interaction may be explained by the absence of a stroking condition and distance interaction in OAs. Nevertheless, additional studies are needed to investigate whether it is possible to strengthen the FBI for older adults, and if this in turn would lead to a transfer of PPS to the avatar also in older adults. An additional limitation is that, guided by results observed in YAs in Experiment 2, as well as the practical consideration of keeping the test duration feasible for older participants, we did not include looming stimuli in Experiment 3. This prevents a full understanding of whether PPS in older adults may adapt to VR and if it is comparable to virtual PPS representations in young adults. Existing data show a distance-dependent multisensory effect for looming audio-tactile stimulation in physical environments in healthy elderly individuals, indicating a functioning PPS representation (Sorrentino et al. 2021; see also Bassolino et al. 2022 for brain damaged older participants). However, whether a similar pattern of multisensory facilitation for looming stimuli is present in VR between young and older adults remains an open question for future research.

It is also worth considering whether age-related changes in motion perception may have influenced the age differences in RTF observed in the visuo-tactile task. Although Experiment 3 included only receding stimuli and therefore did not require participants to discriminate between visual stimulus direction, motion perception is known to decline with age (Billino and Pilz 2019). However, older adults exhibit deficits primarily in tasks requiring integration of motion over noise and retain accurate motion perception for fast and high contrast stimuli (Billino and Pilz 2019), as well as motion cues with a high signal-to-noise ratio, even at brief presentation durations like the 400 ms used in our setup (Pilz et al. 2017). To support effective motion perception in both age groups, our visual stimuli were optimized to meet these conditions: they were high contrast (a bright green ball), presented in isolation without noise, and moved relatively fast (33.5 cm/s) on a consistent diagonal trajectory. This visual stimulus design, supported by evidence from the existing literature, address potential concerns about age-related perceptual limitations. Nonetheless, when employing the visuo-tactile task for age comparisons, it is important to carefully consider motion parameters to account for age-related perceptual changes.

Another potential concern is the subjective nature of the ownership ratings in our study. It could be that the observed age differences in ownership ratings partly reflect differences in response tendencies across age groups. However, it should be noted that we also conducted an age comparison of the within-subject differences between ownership ratings in the synchronous and asynchronous condition. This comparison of within-subject differences should be less affected by individual response tendencies, since such tendencies should influence ratings in both stroking conditions similarly. To further strengthen the interpretation of ownership results, future research could incorporate more objective measures alongside subjective reports. For instance, physiological responses such as a decrease in skin temperature of the real body during the FBI, or an increase in galvanic skin response when a threatening stimulus approaches the avatar, can serve as reliable indicators of ownership experience (for a review of current methods used to assess embodiment and its subcomponents see Guy et al. 2023). These objective markers could provide converging evidence for age-related differences in ownership and would enhance the robustness of observed results.

Additionally, it is important to note that we used only one avatar per gender and age group. This is particularly relevant given evidence that greater visual realism and personalization of avatars can enhance avatar embodiment (Salagean et al. 2023). The weaker ownership ratings observed in older adults could therefore partly reflect a lower perceived resemblance to the avatars used for this group. The challenge of creating realistic, individualized avatars remains a general limitation in VR research, largely due to current technical constraints. However, emerging methods for generating human digital twins (e.g., Lin et al. 2024) offer promising and accessible solutions. Applying such digital twins in FBI paradigms could improve perceived resemblance and potentially enhance the sense of ownership.

In conclusion, this study offers a novel finding on the suggested relationship between PPS and avatar embodiment and indicates that PPS in young adults can transfer to an embodied location in VR that is separate from the physical body. This is significant for understanding the fundamental mechanisms of PPS, while also providing guidance for the design of virtual applications. The absence of this effect in older adults may derive from a reduced susceptibility to the FBI in old age. However, additional studies are needed to build on our findings and address the limitations of our study. To support the development of age-inclusive virtual technology, as well as to better understand the effect of aging on embodiment, it is important that these age-related differences are further elucidated.

## Electronic supplementary material

Below is the link to the electronic supplementary material.

Supplementary Material 1

## Data Availability

The datasets analyzed during the current study are available in the following Open Science Framework repository link: https://osf.io/wvryn/?view_only=146ad6c5155f421b9857363bb0809342.
