# Supplementary material for: Transfer of peripersonal space to a virtual body in young adults and exploration of adult age differences
Source: Exp Brain Res. 2025 Jul 25;243(8):189. doi: 10.1007/s00221-025-07132-6 (PMC12296863; doi:10.1007/s00221-025-07132-6)
Supplement: Supplementary file 1 — Supplementary Material 1 [file 221_2025_7132_MOESM1_ESM.docx]

**Supplementary Materials**

**Transfer of peripersonal space to a virtual body in young adults and exploration of adult age differences**

Dariusz O’Leary^1,2,*^, Yichen Fan^1,3^, Ina Schattenberg^2^, Andrea Serino^4^ & Shu-Chen Li^1,2,5,*^

1. 6G-life Research Hub, TU Dresden, Dresden, Germany
2. Chair of Lifespan Developmental Neuroscience, Faculty of Psychology, TU Dresden, Dresden, Germany
3. Chair of Industrial Design Engineering, TU Dresden, Dresden, Germany
4. MySpace Lab, Department of Clinical Neuroscience, University Hospital of Lausanne, Lausanne, Switzerland
5. Centre for Tactile Internet with Human-in-the-Loop, TU Dresden, Dresden, Germany

^*^Corresponding authors: dariusz.oleary@tu-dresden.de, shu-chen.li@tu-dresden.de

**Table S1**

*Embodiment Questionnaire in English*

| **Instructions** |
| --- |
| In reference to the preceding experimental block, please indicate whether there were moments for which you would agree with the following statements (from "*strongly disagree (1)*" to "*strongly agree (7)*"). |
| **Items** |
| - 1. I felt out of my body.   2. I felt as if my (real) body were drifting toward the virtual body or as if the virtual body were drifting toward my (real) body.   3. It felt as if my (real) body were turning into an “avatar” body.   4. At some point it felt as if my (real) body was starting to take on the posture or shape of the virtual body that I saw.   5. I felt like I was wearing different clothes from when I came to the laboratory.   6. I felt as if my body had changed.   7. I felt that my own body could be affected by the virtual world.   8. I felt as if the virtual body was my body.   9. At some point it felt that the virtual body resembled my own (real) body, in terms of shape, skin tone or other visual features.   10. I felt as if my body was located where I saw the virtual body.   11. I felt like I could control the virtual body as if it was my own body.   12. It seemed as if I felt the touch of the controller in the location where I saw the virtual body touched.   13. It seemed as if the touch I felt was caused by the virtual controller touching the virtual body.   14. It seemed as if my body was touching the virtual controller.   15. I felt as if I was in the virtual room.   16. I felt nauseous. |
| **Likert Scale** |

| strongly disagree | disagree | somewhat disagree | neither agree nor disagree | somewhat agree | agree | strongly  agree |
| --- | --- | --- | --- | --- | --- | --- |
| (1) | (2) | (3) | (4) | (5) | (6) | (7) |

*Note.* The original questionnaire contains 16 questions, of which we removed two, as one referred to movement of the avatar body, which was not included in our experiment (R3 in Peck and Gonzalez-Franco (2021)), and one was consistently found to be confusing by pilot participants (R8 in Peck and Gonzalez-Franco (2021)). Moreover, we added a question assessing the feeling of presence in the virtual room (Q15), as well as a question to control for nausea (Q16). Participants responded to the 16 questions using a 7-point Likert scale ranging from “1 = strongly disagree” to “7 = strongly agree”.

**Table S2**

*Embodiment Questionnaire Translated into German*

| **Instruktionen** |
| --- |
| Geben Sie bitte in Bezug auf den vorhergehenden experimentellen Block an, ob es Momente gab, für die Sie den folgenden Aussagen zustimmen würden (von "*stimme überhaupt nicht zu (1)*" zu "*stimme vollkommen zu (7)*"). |
| **Fragen** |
| - 1. Ich hatte das Gefühl, nicht mehr in meinem Körper zu sein.   2. Ich hatte das Gefühl, als würde mein (realer) Körper auf den virtuellen Körper zudriften oder als würde der virtuelle Körper auf meinen (realen) Körper zudriften.   3. Ich hatte das Gefühl, als würde sich mein (realer) Körper in einen „Avatar“-Körper verwandeln.   4. Irgendwann hatte ich das Gefühl, dass mein (realer) Körper anfing, die Haltung oder Form des virtuellen Körpers anzunehmen.   5. Ich hatte das Gefühl, dass ich andere Kleidung trug als zu dem Zeitpunkt, als ich das Labor betrat.   6. Ich hatte das Gefühl, dass sich mein Körper verändert hatte.   7. Ich hatte das Gefühl, dass mein eigener Körper von der virtuellen Welt beeinflusst werden konnte.   8. Ich hatte das Gefühl, dass der virtuelle Körper mein Körper ist.   9. Irgendwann hatte ich das Gefühl, dass der virtuelle Körper meinem eigenen (realen) Körper ähnelt, in Bezug auf Form, Hautfarbe oder andere visuelle Merkmale.   10. Ich hatte das Gefühl, dass sich mein Körper dort befand, wo ich den virtuellen Körper sah.   11. Ich hatte das Gefühl, dass ich den virtuellen Körper kontrollieren konnte, als wäre es mein eigener Körper.   12. Es schien, als ob ich die Berührung des Controllers an der Stelle spürte, an der ich den virtuellen Körper berührt sah.   13. Es schien, als ob die Berührung, die ich spürte, durch die Berührung des virtuellen Controllers mit dem virtuellen Körper verursacht wurde.   14. Es schien, als ob mein Körper den virtuellen Controller berührte.   15. Ich hatte das Gefühl, mich in dem virtuellen Raum zu befinden.   16. Mir war übel. |
| **Likert Skala** |

| stimme überhaupt nicht zu | stimme nicht zu | stimme eher nicht zu | weder noch | stimme eher zu | stimme zu | stimme vollkommen zu |
| --- | --- | --- | --- | --- | --- | --- |
| (1) | (2) | (3) | (4) | (5) | (6) | (7) |

*Note.* The original questionnaire contains 16 questions, of which we removed two, as one referred to movement of the avatar body, which was not included in our experiment (R3 in Peck and Gonzalez-Franco (2021)), and one was consistently found to be confusing by pilot participants (R8 in Peck and Gonzalez-Franco (2021)). Moreover, we added a question assessing the feeling of presence in the virtual room (Q15), as well as a question to control for nausea (Q16). Participants responded to the 16 questions using a 7-point Likert scale ranging from “1 = stimme überhaupt nicht zu” to “7 = stimme vollkommen zu”.

**Calculation of Ownership Score**

Following the method suggested by Peck and Gonzalez (2021), we calculated the Ownership score as follows (see corresponding items in Table S1 and Table S2 above):

Ownership = (Q4 + Q8 + Q9 + Q10 + Q11 + Q12)/6

**Covariate Analyses for Experiment 3**

To control for age differences in tactile perception, participants in Experiment 3 completed an initial tactile threshold test in which participants were presented with a tactile stimulus that increased in intensity from 5 % up to 100 % in steps of 5 %. Participants were required to indicate if they perceived the tactile stimulus after each step and the first step at which they gave three positive responses in sequence, was taken as their tactile threshold. The tactile threshold for one YA participant could not be collected due to a technical failure during testing. Additionally, to be able to consider age differences in familiarity with VR headsets and time spent gaming in Experiment 3, participants responded to a follow-up VR and gaming experience questionnaire (see Table S3 for the original German version and Table S4 for an English translation), for which we had complete data for 25 out of 26 OAs and 23 out of 27 YAs.

We compared the log-transformed tactile thresholds between age groups and found significantly higher tactile thresholds in OAs compared to YAs (*t*(48.79) = 2.69, *p* = .010, *d* = 0.75). Moreover, we compared the total time spent using VR headsets in the past, as well as the average time per week spent gaming between age groups. While we found no difference between age groups in the total time spent using VR headsets (*W* = 270, *p* = .668, *r* = 0.06), we did find a significantly greater average time spent gaming per week for YAs compared to OAs (*W* = 169, *p* = .007, *r* = 0.39).

We therefore reran the three-way ANOVA on data from Experiment 3, with the within-subject factors distance (six levels: 0.3 m, 0.6 m, 0.9 m, 1.2 m, 1.5 m, 1.8 m) and stroking condition (two levels: synchronous, asynchronous) and the between-subject factor age (two levels: young, old), once with tactile threshold as a covariate (*tthresh*), and once with time spent gaming as a covariate (*gaming*). Other than the main effect of age being marginally significant for the ANOVA with time spent gaming as a covariate (*p_gaming_* = .052), the same effects were significant for the two ANOVAs with covariates and the ANOVA without covariates reported in the main text. In all three ANOVAs we found a significant main effect of distance (*F*(3.56, 181.48) = 7.59, *p* < .0001, *η_p_^2^* = 0.13; *F_tthresh_* (3.51, 172.23) = 7.59, *p_tthresh_* < .0001, *η_p_^2^_tthresh_* = 0.13; *F_gaming_* (3.59, 161.57) = 3.98, *p_gaming_* = .006, *η_p_^2^_gaming_* = 0.08), a (marginally) significant main effect of age (*F*(1, 51) = 10.22, *p* = .002, *η_p_^2^* = 0.17; *F_tthresh_* (1, 49) = 6.45, *p_tthresh_* = .014, *η_p_^2^_tthresh_* = 0.12; *F_gaming_* (1, 45) = 4.00, *p_gaming_* = .052, *η_p_^2^_gaming_* = 0.08), and a significant interaction between distance and age (*F*(3.56, 181.48) = 4.88, *p* = .001, *η_p_^2^* = 0.09; *F_tthresh_*(3.51, 172.23) = 4.22, *p_tthresh_* = .004, *η_p_^2^_tthresh_* = 0.08; *F_gaming_*(3.59, 161.57) = 5.14, *p_gaming_* = .001, *η_p_^2^_gaming_* = 0.10). These results confirm that these covariates do not affect the main patterns and interpretations of results observed in Experiment 3.

**Table S3**

*Gaming and VR Experience Questionnaire in German*

| **Fragen** |
| --- |
| - 1. Während der letzten 12 Monate, wie viel Zeit haben Sie im Durchschnitt pro Woche damit verbracht Videospiele zu spielen?  \| niemals \| weniger als 1 Stunde \| zwischen 1 und 3 Stunden \| zwischen 3 und 5 Stunden \| zwischen 5 und 10 Stunden \| mehr als 10 Stunden \| \| --- \| --- \| --- \| --- \| --- \| --- \| \|  \|  \|  \|  \|  \|  \|  - 1. Welche Art von Videospiel haben Sie in den letzten 12 Monaten am meisten gespielt (mehrere Antworten möglich)? - Shooter (Call of Duty, Halo, Overwatch, Counterstrike, Apex Legends ...) - Rollenspiele (Elden Ring, The Witcher, Elder Scrolls, Starfield ...) - Sport (FIFA, Mario Kart, Forza ...) - Platformer (Super Mario, Ratchet & Clank, Ori and the Blind Forest ...) - Strategie/Puzzle (Starcraft, Civilization, League of Legends ...) - Musik (Guiter Hero, Beat Saber, Just Dance ...) - Andere (Handyspiele, Browserspiele ...)   1. **Bevor** Sie uns im Labor besucht haben, besaßen Sie eine eigene Virtual Reality (VR) Brille (z.B. die gleiche oder eine ähnliche virtuelle Brille, zu der, die Sie bei uns im Labor anhatten)?  \| ja \| nein \| \| --- \| --- \| \|  \|  \|  - 1. **Bevor** Sie uns im Labor besucht haben, wie viel Zeit haben Sie insgesamt damit verbracht eine Virtual Reality (VR) Brille zu benutzen?  \| niemals \| weniger als 1 Stunde \| zwischen 1 und 3 Stunden \| zwischen 3 und 5 Stunden \| zwischen 5 und 10 Stunden \| zwischen 10 und 30 Stunden \| mehr als 30 Stunden \| \| --- \| --- \| --- \| --- \| --- \| --- \| --- \| \|  \|  \|  \|  \|  \|  \|  \|  - 1. **Nachdem** Sie uns im Labor besucht haben, haben Sie sich eine eigene Virtual Reality (VR) Brille gekauft?  \| ja \| nein \| \| --- \| --- \| \|  \|  \|  - 1. **Nachdem** Sie uns im Labor besucht haben, wie viel Zeit haben Sie insgesamt damit verbracht eine Virtual Reality (VR) Brille zu benutzen?  \| niemals \| weniger als 1 Stunde \| zwischen 1 und 3 Stunden \| zwischen 3 und 5 Stunden \| zwischen 5 und 10 Stunden \| zwischen 10 und 30 Stunden \| mehr als 30 Stunden \| \| --- \| --- \| --- \| --- \| --- \| --- \| --- \| \|  \|  \|  \|  \|  \|  \|  \| \|  \|  \|  \|  \|  \|  \|  \| |

*Note.* Q2 was only shown if any option other than “niemals” was chosen in Q1. The average time spent gaming covariate was based on responses to Q1, whereas the total time spent using VR headsets was based on responses to Q4.

**Table S4**

*Gaming and VR Experience Questionnaire Translated into English*

| **Items** |
| --- |
| - 1. Over the last 12 months, how much time on average did you spend per week playing video games?  \| never \| less than 1 hour \| between 1 and 3 hours \| between 3 and 5 hours \| between 5 and 10 hours \| more than 10 hours \| \| --- \| --- \| --- \| --- \| --- \| --- \| \|  \|  \|  \|  \|  \|  \|  - 1. What type of video game did you play the most in the last 12 months (multiple answers possible)? - Shooter (Call of Duty, Halo, Overwatch, Counterstrike, Apex Legends ...) - Role-Playing (Elden Ring, The Witcher, Elder Scrolls, Starfield ...) - Sport (FIFA, Mario Kart, Forza ...) - Platformer (Super Mario, Ratchet & Clank, Ori and the Blind Forest ...) - Strategy/Puzzle (Starcraft, Civilization, League of Legends ...) - Music (Guiter Hero, Beat Saber, Just Dance ...) - Other (Mobile Games, Browser Games ...)   1. **Before** you visited us in the lab, did you have your own virtual reality (VR) headset (e.g. the same or similar virtual headset to the ones you wore in our lab)  \| yes \| no \| \| --- \| --- \| \|  \|  \|  - 1. **Before** you visited us in the lab, how much time in total did you spend using virtual reality (VR) headsets?  \| never \| less than 1 hour \| between 1 and 3 hours \| between 3 and 5 hours \| between 5 and 10 hours \| between 10 and 30 hours \| more than 30 hours \| \| --- \| --- \| --- \| --- \| --- \| --- \| --- \| \|  \|  \|  \|  \|  \|  \|  \|  - 1. **After** you visited us in the lab, did you buy your own virtual reality (VR) headset?  \| yes \| no \| \| --- \| --- \| \|  \|  \|  - 1. **After** you visited us in the lab, how much time in total did you spend using virtual reality (VR) headsets?  \| never \| less than 1 hour \| between 1 and 3 hours \| between 3 and 5 hours \| between 5 and 10 hours \| between 10 and 30 hours \| more than 30 hours \| \| --- \| --- \| --- \| --- \| --- \| --- \| --- \| \|  \|  \|  \|  \|  \|  \|  \| \|  \|  \|  \|  \|  \|  \|  \| |

*Note.* Q2 was only shown if any option other than “never” was chosen in Q1. The average time spent gaming covariate was based on responses to Q1, whereas the total time spent using VR headsets was based on responses to Q4.

**Supplementary References**

Peck TC, Gonzalez-Franco M (2021) Avatar embodiment. A standardized questionnaire. Front Virtual Real 1:1–12. https://doi.org/10.3389/frvir.2020.575943
